# Development of a new fusion-enhanced oncolytic immunotherapy platform based on herpes simplex virus type 1

**DOI:** 10.1186/s40425-019-0682-1

**Published:** 2019-08-10

**Authors:** Suzanne Thomas, Linta Kuncheria, Victoria Roulstone, Joan N. Kyula, David Mansfield, Praveen K. Bommareddy, Henry Smith, Howard L. Kaufman, Kevin J. Harrington, Robert S. Coffin

**Affiliations:** 1 Replimune Inc, 18 Commerce Way, Woburn, MA 01801 USA; 2 Institute for Cancer Research, London, UK; 3 Massachusetts General Hospital, Boston, MA USA

**Keywords:** Oncolytic viruses, Herpes simplex-1, Immunotherapy, Anenestic effect

## Abstract

**Background:**

Oncolytic viruses preferentially replicate in tumors as compared to normal tissue and promote immunogenic cell death and induction of host systemic anti-tumor immunity. HSV-1 was chosen for further development as an oncolytic immunotherapy in this study as it is highly lytic, infects human tumor cells broadly, kills mainly by necrosis and is a potent activator of both innate and adaptive immunity. HSV-1 also has a large capacity for the insertion of additional, potentially therapeutic, exogenous genes. Finally, HSV-1 has a proven safety and efficacy profile in patients with cancer, talimogene laherparepvec (T-VEC), an oncolytic HSV-1 which expresses GM-CSF, being the only oncolytic immunotherapy approach that has received FDA approval. As the clinical efficacy of oncolytic immunotherapy has been shown to be further enhanced by combination with immune checkpoint inhibitors, developing improved oncolytic platforms which can synergize with other existing immunotherapies is a high priority. In this study we sought to further optimize HSV-1 based oncolytic immunotherapy through multiple approaches to maximize: (i) the extent of tumor cell killing, augmenting the release of tumor antigens and danger-associated molecular pattern (DAMP) factors; (ii) the immunogenicity of tumor cell death; and (iii) the resulting systemic anti-tumor immune response.

**Methods:**

To sample the wide diversity amongst clinical strains of HSV-1, twenty nine new clinical strains isolated from cold sores from otherwise healthy volunteers were screened across a panel of human tumor cell lines to identify the strain with the most potent tumor cell killing ability, which was then used for further development. Following deletion of the genes encoding ICP34.5 and ICP47 to provide tumor selectivity, the extent of cell killing and the immunogenicity of cell death was enhanced through insertion of a gene encoding a truncated, constitutively highly fusogenic form of the envelope glycoprotein of gibbon ape leukemia virus (GALV-GP-R^−^). A number of further armed derivatives of this virus were then constructed intended to further enhance the anti-tumor immune response which was generated following fusion-enhanced, oncolytic virus replication-mediated cell death. These viruses expressed GMCSF, an anti-CTLA-4 antibody-like molecule, CD40L, OX40L and/or 4-1BB, each of which is expected to act predominantly at the site and time of immune response initiation. Expression of these proteins was confirmed by ELISA and/or western blotting. Immunogenic cell death was assessed by measuring the levels of HMGB1 and ATP from cell free supernatants from treated cells, and by measuring the surface expression of calreticulin. GALV-GP-R^−^ mediated cell to cell fusion and killing was tested in a range of tumor cell lines in vitro. Finally, the in vivo therapeutic potential of these viruses was tested using human A549 (lung cancer) and MDA-MB-231(breast cancer) tumor nude mouse xenograft models and systemic anti-tumor effects tested using dual flank syngeneic 4434 (melanoma), A20 (lymphoma) mouse tumor models alone and in combination with a murine anti-PD1 antibody, and 9 L (gliosarcoma) tumors in rats.

**Results:**

The twenty nine clinical strains of HSV-1 isolated and tested demonstrated a broad range of tumor cell killing abilities allowing the most potent strain to be identified which was then used for further development. Oncolytic ability was demonstrated to be further augmented by the expression of GALV-GP-R^−^ in a range of tumor cell lines in vitro and in mouse xenograft models in nude mice. The expression of GALV-GP-R^−^ was also demonstrated to lead to enhanced immunogenic cell death in vitro as confirmed by the increased release of HMGB1 and ATP and increased levels of calreticulin on the cell surface. Experiments using the rat 9 L syngeneic tumor model demonstrated that GALV-GP-R^−^ expression increased abscopal uninjected (anenestic) tumor responses and data using mouse 4434 tumors demonstrated that virus treatment increased CD8+ T cell levels both in the injected and uninjected tumor, and also led to increased expression of PD-L1. A combination study using varying doses of a virus expressing GALV-GP-R^−^ and mGM-CSF and an anti-murine PD1 antibody showed enhanced anti-tumor effects with the combination which was most evident at low virus doses, and also lead to immunological memory. Finally, treatment of mice with derivatives of this virus which additionally expressed anti-mCTLA-4, mCD40L, m4-1BBL, or mOX40L demonstrated enhanced activity, particularly in uninjected tumors.

**Conclusion:**

The new HSV-1 based platform described provides a potent and versatile approach to developing new oncolytic immunotherapies for clinical use. Each of the modifications employed was demonstrated to aid in optimizing the potential of the virus to both directly kill tumors and to lead to systemic therapeutic benefit. For clinical use, these viruses are expected to be most effective in combination with other anti-cancer agents, in particular PD1/L1-targeted immune checkpoint blockade. The first virus from this program (expressing GALV-GP-R^−^ and hGM-CSF) has entered clinical development alone and in combination with anti-PD1 therapy in a number of tumor types (NCT03767348).

**Electronic supplementary material:**

The online version of this article (10.1186/s40425-019-0682-1) contains supplementary material, which is available to authorized users.

## Introduction

Oncolytic immunotherapy has shown single agent clinical activity and synergy with immune checkpoint blockade. However, not all patients respond, and most of the clinical experience has been in melanoma. With the objective of maximally activating a patient’s immune system against their own cancer to enhance synergy with anti-PD1/L1 blockade, we have developed a new oncolytic immunotherapy platform based on herpes simplex virus type 1 (HSV-1). This has the dual objectives of robustly killing tumor to provide abundant release of tumor antigens, and potently activating the immune system against these tumor antigens once released. To augment the natural ability of HSV-1 to kill tumors and activate anti-tumor immunity, the viruses developed are armed with therapeutic genes with the expectation that ‘arming’ will be essential to maximizing clinical activity. Initially, we sampled the genetic variation between strains of HSV-1 by screening twenty nine new clinical strains isolated from volunteers who suffer from cold sores across a panel of human tumor cell lines to identify the strain to be developed. This strain (RH018A) was then engineered for oncolytic use by deletion of the genes encoding ICP34.5 to reduce pathogenicity, deleting the ICP47 encoding gene to enhance viral and tumor antigen presentation by major histocompatibility complex-I (MHC-I), and inserting a gene encoding a potent fusogenic glycoprotein derived from gibbon ape leukemia virus (GALV-GP-R^−^). Expression of GALV-GP-R^−^ caused increased immunogenic cell death, assessed by the release of danger-associated molecular pattern factors, activated anti-tumor immunity, and enhanced systemic therapeutic activity against rat and murine tumors in vivo*.* Additionally, the virus induced expression of PD-L1, and demonstrated enhanced activity in combination with PD-1 blockade. A virus expressing GALV-GP-R^−^ and hGM-CSF is currently in a Phase 1/2 clinical trial (NCT03767348). Further viruses were constructed based on this virus which additionally express an anti-CTLA-4 antibody or immune co-stimulatory pathway activating ligands, each of which is expected to act at the site and time of immune response initiation in the injected tumor and draining lymph nodes. These viruses demonstrated further increased activity in mice, particularly an enhanced anenestic effect. This data supports the potential for improved therapeutic activity of this new oncolytic immunotherapy platform and demonstrate its use to express immune modulatory proteins which may provide a generalized strategy to improve therapy for patients with cancer. There have been significant advances in the immunotherapy of cancer, most notably through the clinical development of immune checkpoint inhibitors targeting cytotoxic T lymphocyte antigen 4 (CTLA-4) and the programmed cell death 1 (PD-1)/PD-1 ligand (PD-L1) pathway [1, 2]. While durable clinical responses have been observed across numerous solid and hematologic malignancies, many tumors do not respond or develop resistance over time [3]. The absence of tumor-specific T cells within the tumor microenvironment appears to be an important feature associated with innate and acquired resistance to checkpoint blockade. New strategies that can induce anti-tumor immune responses with which anti-PD-1/L1 therapy can synergize, reverse the immune-deficient tumor microenvironment, and which can re-establish tumor sensitivity to systemic anti-PD-1/L1 therapy are therefore needed. One promising approach is virus-based oncolytic immunotherapy [4]. Oncolytic viruses preferentially replicate in tumors as compared to normal tissue, and promote immunogenic cell death and induction of host systemic anti-tumor immunity. The oncolytic immunotherapy approach has been clinically validated as demonstrated by the U.S. Food and Drug Administration (FDA) and European Medicines Agency (EMA) approval of talimogene laherparepvec (T-VEC), an oncolytic herpes simplex virus type 1 (HSV-1) encoding GM-CSF, for the treatment of advanced melanoma in 2015 [5]. The phase 3 clinical trial which led to the approval of T-VEC demonstrated a 26.4% objective response rate, and a 10.8% complete response rate (rising to 17% at the time of the final analysis [Amgen ODAC presentation May 2015] [6]), in a 436-patient phase 3 study in patients with both previously treated and previously untreated Stage IIIb-IVM1c disease [5].

The therapeutic potential of T-VEC can be further enhanced by combination with immune checkpoint inhibitors. In a small phase 1 trial in patients with melanoma, T-VEC in combination with pembrolizumab resulted in a 62% response rate and 33% complete response rate [7]. Similarly promising response rates (> 50%) have also been seen in other small studies with either ipilimumab or pembrolizumab in combination with other oncolytic viruses, such as Cavatak (an oncolytic Coxsackievirus) or HF10 (another oncolytic HSV-1) [4]. Data have also been reported from a 200-patient randomized controlled phase 2 clinical trial with T-VEC combined with ipilimumab compared to ipilimumab alone, where more than a doubling of the response rate was seen in the combination arm [8]. While these studies were all in melanoma, it is important to note that none reported significant additional toxicity compared to that expected with either agent alone. Based on the favorable therapeutic window for T-VEC and other oncolytic viruses, there has been considerable interest in optimizing the oncolytic immunotherapy strategy and using such agents as part of a rational combination regimen in patients with solid cancers.

It is now generally accepted that patients responding to immunotherapy need to have tumors that are immunologically ‘hot’, i.e. have a T cell-inflamed phenotype, although the specific mechanisms that regulate T cell recruitment into established tumors are incompletely understood [9]. Additional factors that favor immune-mediated rejection include high mutation burden, presence of pre-existing immune responses to tumor antigens, particularly tumor neoantigens, and expression of a pro-inflammatory gene signature [10]. While a number of approaches are in development aimed at correcting these deficiencies in non-responsive patients, oncolytic immunotherapies may have particular promise for this purpose as they kill tumors in a highly inflammatory context. This effect is highly immunogenic, including activation of both innate and adaptive immunity, with the potential to create a vaccine “in situ” within the patient against their own cancer. The local production of type 1 interferons induced by oncolytic viruses also results in increased expression of several immune regulatory proteins, including MHC class I and PD-L1 [4].

Thus, oncolytic immunotherapy appears to be particularly well suited for combination strategies with immune checkpoint blockade. We sought to further optimize the approach by maximizing (i) the extent of tumor cell killing, augmenting the release of tumor antigens and danger-associated molecular pattern (DAMP) factors; (ii) the immunogenicity of tumor cell death; and (iii) the resulting systemic anti-tumor immune response. While a range of viral species were considered for development, HSV-1 was selected for several reasons. First, HSV-1 is a very lytic DNA virus; it infects human tumor cells broadly, and when ICP34.5 is deleted exhibits preferential replication in neoplastic tissue. Second, HSV-1 kills mainly by necrosis and activates innate immunity, including through the cGAS/STING pathway. Third, HSV-1 has a large capacity for the insertion of additional, potentially therapeutic, exogenous genes. Finally, HSV-1 has a proven safety and efficacy profile in patients with cancer. While intravenous administration was also considered, an intratumoral approach, i.e. local administration providing systemic immune-based benefit, was selected based on prior clinical validation and the considerable, and potentially insurmountable, biological hurdles to effective intravenous dosing [4, 11]. HSV-1 causes cold sores in humans and is widely prevalent in the population, with up to 90% of individuals testing seropositive by the age of 65 [12]. However, substantial natural variation might be expected amongst clinical strains of HSV-1 (i.e. as sampled from individuals suffering from cold sores) with respect to evolved biological properties such as virulence. This natural variation might also translate into differences in non-evolved properties, such as the ability to infect and kill human tumor cells. Based on the hypothesis that prototypical ‘laboratory’ strains of HSV-1, such as Strain 17+, KOS or Strain F may have become attenuated through extended serial passage or may otherwise not be optimal strains for cancer therapy, T-VEC was initially derived from a clinical strain of HSV-1 after comparing two clinical isolates to Strain 17+. Both of the clinical strains were superior for human tumor cell killing compared to Strain 17+, and the most promising of the two, Strain JS1, was chosen and engineered into T-VEC [13].

In this report, we describe the generation and characterization of a new HSV-1-based oncolytic immunotherapy platform which utilizes a strain of HSV-1 selected from twenty nine newly isolated clinical strains on the basis of increased oncolytic activity in vitro. This was then engineered for tumor selectivity and to express a potent fusogenic membrane glycoprotein (GALV-GP-R^−^) to increase the extent and immunogenicity of tumor cell death. Various fusogenic proteins including from measles virus and various retroviruses have previously been tested in replicative and non-replicative virus-mediated gene therapy approaches to the treatment of cancer in pre-clinical models [14], including when delivered by oncolytic versions of HSV [15]. Fusogenic cell death has also previously been demonstrated to be highly immunogenic [14]. Genes encoding GM-CSF, an anti-CTLA-4 antibody-like molecule and a number of immune co-stimulatory pathway-activating ligands were then inserted, intending to further enhance the systemic, immune-mediated, anti-tumor effects achieved.

## Methods

### Assessment of GALV-GP-R^−^ mediated fusion

The cell lines used for the fusion assays were A549 (ECACC 91072201), HT29 (ECACC 91072201), HT1080 (ECACC 85111505), MDA-MB-231 (ECACC 92020424), miaPaCa-2 (ECACC 85062806) and SK-mel-28 (ATCC® HTB-72™). The monolayers were infected using a range of multiplicity of infection (MOI) from 0.01 to 0.0001. The infected cell monolayers were observed for GFP expression at 24 h. and 48 h. post-infection and then fixed and stained with crystal violet.

### Western blots and ELISA

For detection of anti-CTLA-4 expressed from Virus 27, supernatant was used from BHK cells infected at MOI =1 in serum-free mediuma for 24 h. The proteins were separated on 10–20% sodium dodecyl polyacrylamide gel (Thermo Fisher CAT No: XP10200BOX) and transferred to polyvinylidene difluoride membrane (Life Technologies Cat No: LC2005). The membrane was probed with goat anti-mouse IgG1 heavy chain (alkaline phosphatase) (Abcam Cat No: ab97237). BCIP®/NBT Liquid Substrate System (Sigma Aldrich Cat No: B1911) was used for the detection.

For detection of CD40L, 4-1BBL and OX40L from Viruses 32, 33 and 35, respectively, BHK cells were infected at MOI =1 for 24 h. To confirm expression of 4-1BBL from Virus 33, microplates were coated with the capture antibody (0.5μg/ml, R&D Systems Cat No:-AF1246) and incubated overnight at 4 °C. Following blocking, standards (R&D Systems Cat No 1256-4 L, 40 ng/ml- 0.63 ng/ml) and samples were added and incubated at 37 °C. The wells were then probed with anti-mouse 41BBL (Bioxcell Cat No: BE0110) after which HRP Tagged antibody (Sigma Aldrich Cat No: A5795) was added and incubated for 1 h. TMB was added and incubated for 5 mins and sulphuric acid was added to stop the reaction. The plates were read at 450 nm. ELISA for CD40L (Abcam Cat No: ab119517) and OX40L (Thermo Fisher Cat No: EMTNFSF4) was performed using kits as per the manufacturer’s instructions.

### ATP release

Cells were plated at 2 × 10^5^ cells per well in 1 mL, in 12-well plates, and incubated overnight. Cells were then infected with Virus 23 or Virus 17 the following day. Twenty-four and 48 h after treatment, cell supernatants were collected and centrifuged at 2000 rpm for 4 mins. Cell-free supernatants were then measured for ATP by CellTiter-Glo Luminescent Cell Viability Assay (CTG, Promega, UK). Fifty microliter of CTG was added per 200 uL sample and incubated for 10 min. Luminescence was measured on a Victor 2 V plate reader (Perkin Elmer).

### High mobility group box 1 protein (HMGB1) release

Cells were plated at 2 × 10^5^ cells per well in 1 mL, in 12-well plates, and incubated overnight. Cells were infected with Virus 23 or Virus 17 the following day. Forty-eight h after treatment, cell supernatants were collected and centrifuged at 2000 rpm for 4 mins. Cell-free supernatants were then measured for HMGB1 by an ELISA Assay (IBL International GmbH Cat No: ST51011) as per the manufacturer’s instructions.

### Cell surface calreticulin expression

Cells were plated at 2 × 10^5^ cells per well in 1 mL, in 12-well plates, and incubated overnight. Cells were infected with Virus 23 or Virus 17 the following day at various MOI. Forty-eight h after treatment, un-permeabilized samples were stained with viability dye (Thermo Fisher Cat No: 65–0865-14), with anti-calreticulin antibody (Abcam Cat No: ab92516), or isotype control antibody (Abcam Cat No: ab172730), and flow cytometry was performed. Surface calreticulin expression was shown as median fluorescence intensity (MFI). Data was analyzed using FlowJo software.

### In vivo efficacy testing

Bilateral mouse A20 lymphoma tumors were grown in of Balb/c mice or human A549 or MDA-MB-231 tumors grown in the right flanks of Balb/c nude mice until average tumor diameters were > 5 mm. Right flank tumors were then injected 3 times (every other day) with the indicated virus and dose in 50 μl or with vehicle (PBS) and tumor diameters were then followed. For experiments in rats, rat 9 L glioma tumors were grown in the left and right flanks of Fischer 344 rats until tumors were 0.75-1 cm in diameter and right flank tumors then dosed 5x (approximately every other day) with the indicated virus at a dose of 5 × 10^6^ pfu in 50 μl or with vehicle and tumor diameters then followed. For experiments in combination with anti-murine PD1, clone RMP1–14 (BioXCell) was given by the intraperitoneal route at 10 mg/kg every 3 days for a total of 9 doses.

### Vectra staining

Vectra staining was performed on tumors to identify tumor infiltrating immune cells as previoulsy described [16]. Bi-flank 4434 murine melanoma tumours grown in C57BL/6 mice were treated with Virus 16 on days 1, 3 and 5, then collected at day 10 after the first injection, fixed overnight in 10% neutral buffered formalin and then transferred to PBS prior to processing and embedding. Tissue sections were labeled with immunofluorescent stains as follows; CD8 (Cat No: 14–0808-82), CD4 (Cat No:14–9766-82), and foxp3 (Cat No: 14–5773-82), all from eBioscience. Images were then quantified by an automated cell segmentation and phenotyping algorithm, using inForm analysis software (Perkin Elmer). Four thousand four hundred thirty-four cells are a murine melanoma tumor cell line generated in house at The Institute of Cancer Research, London.

### FACS analysis of tumours

C57BL/6 mice were subcutaneously implanted with 4 × 10^6^ 4434 murine melanoma cells suspended in 0.1 mL PBS per flank in a bi-flank model. Tumours were allowed to grow to 6–8 mm and randomized into study groups. The right flank was injected with 5 × 10^6^ plaque forming units (pfu) of Virus 16 in 50 μl or a mock group received formulation buffer (vehicle), given on days 1, 3 and 5. Mice were euthanized when a tumour reached 15 mm in any direction. Tumours were harvested and minced with scissors in digestion mix (0.01% trypsin, 2.5 mg/mL collagenase, 2 mg/mL dispase and 1 mg/mL DNAse in RPMI), and incubated at 37 °C for 30 min. Thereafter, samples were kept on ice. Suspensions were passed through a 70 μm strainer using a 2.5 mL syringe plunger and washed through with RPMI + 5 mM EDTA until only connective tissue remained. Samples were centrifuged at 1500 rpm, for 5 mins at 4 °C) and transferred into a V-well 96 plate. Samples were stained in FACS buffer (PSB + 5% FCS) with the following extracellular antibodies for 30 mins, on ice and protected from light; CD3 (Cat No: 100236), CD4 (Cat No:100406), CD8 (Cat No: 100732) all from BioLegend, PD-L1 (BD Biociences Cat No: 558091), and viability dye (Thermo Fisher Cat No: 65–0865-14). Cells were then washed in FACS buffer and permeabilized and stained with intracellular antibody to foxp3 (Thermo Fisher Cat No: 48–5773-80). Samples were then washed and fixed (1–2% PFA) prior to analysis of tumour infiltrating lymphocytes by flow cytometry. Tumours were weighed on collection and counting beads were added when running the analysis in order to calculate cells per mg of tumour.

### Viral replication

Bi-flank 4434 tumours were collected by dissection, homogenized with 600 μl of serum-free DMEM and centrifuged at 3600 rpm. For 5 mins. Tumor draining lymph nodes corresponding to the injected and contralateral tumors and spleens were collected separately. Supernatants were titred on BHK cells plated at 1 × 10^4^ per well in 96 well plates. Cytopathic effect (CPE) was scored 48–72 h later and viral titer was determined by TCID_50_ assay.

### Viral propagation

All viruses used in the study were propagated using a standard laboratory HSV-1 propagation protocol as described previously [17]. In brief, monolayers of vero cells were infected and virus allowed to seed for 2–3 h after which the monolayer was washed with growth media which was replaced and the cells then left in culture until 100% CPE was observed. Virus was harvested from the supernatant and a standard HSV-1 plaque assay performed to quantify the virus [18].

### Statistical analysis

All statistical analyses were performed using GraphPad Prism software version 7.0a. Tumor growth curves, flow cytometric data and immunohistochemistry counts were compared using an unpaired student’s t test (two-tailed), one-way ANOVA or two-way ANOVA when multiple comparisons were performed. *P* values of less than 0.05 were considered significant. The Figures use the following indications of the level of significance: * = *p* < 0.05, ** = *p* < 0.01, *** = *p* < 0.001, **** = *p* < 0.0001.

## Results

### Selection of the virus strain for development

We sought to extend the hypothesis that strains of HSV-1 with greater oncolytic potential could be derived from a larger sampling of HSV-1 cold sore isolates. To accomplish this, we recruited 126 volunteers who suffered from herpes cold sores between May 2015 and Aug 2015 and, after obtaining informed consent, collected viral swab samples from these volunteers during a recurrent episode of cold sores. Samples were cultured from twenty nine volunteers. These were confirmed to be HSV-1 by anti-HSV-1 antibody staining of infected BHK cell monolayers and then compared against each other across a panel of human tumor cell lines representative of different tumor histologies for their ability to infect and kill rapidly and at low virus dose. As expected, considerable variation in these abilities was seen, with roughly one-third of the isolates being relatively poor, roughly one-third being ‘average’, and nine clearly being more effective than the rest. These nine isolates were then compared more thoroughly across the cell line panel, allowing the generation of a rank order of the top five isolates. Representative data at only an individual time point and MOI in each case are shown in (Additional file 1: Figure S1A). Strain RH018 was chosen as the strain for further development on the basis that it scored either first or second most effective at cell killing on each of the cell lines tested. Compared to a representative ‘average’ strain from the screen, i.e. a strain from the middle-third group (isolate RH065), RH018 yielded approximately a 10-fold increase in cytotoxic potency, as defined by isotoxic efficacy at a 10-fold lower multiplicity of infection (Additional file 1: Figure S1B). Isolate RH018 was sequenced, confirming the presence of the expected HSV-1 encoded genes, but with a variety of small changes across the genome as compared to the originally sequenced prototype HSV-1 genome sequence, Strain 17+ (Genbank NC_001806.2). No attempt was made to determine which of the observed changes, individually or in combination, may be responsible for the improved (as compared to the ‘average’ clinical strain of HSV) tumor cell cell killing properties observed. Based on this screen, the RH018A strain of HSV-1 was, therefore, selected as a foundation for further development.

### Engineering for use as an oncolytic virus

**Fig. 1:**
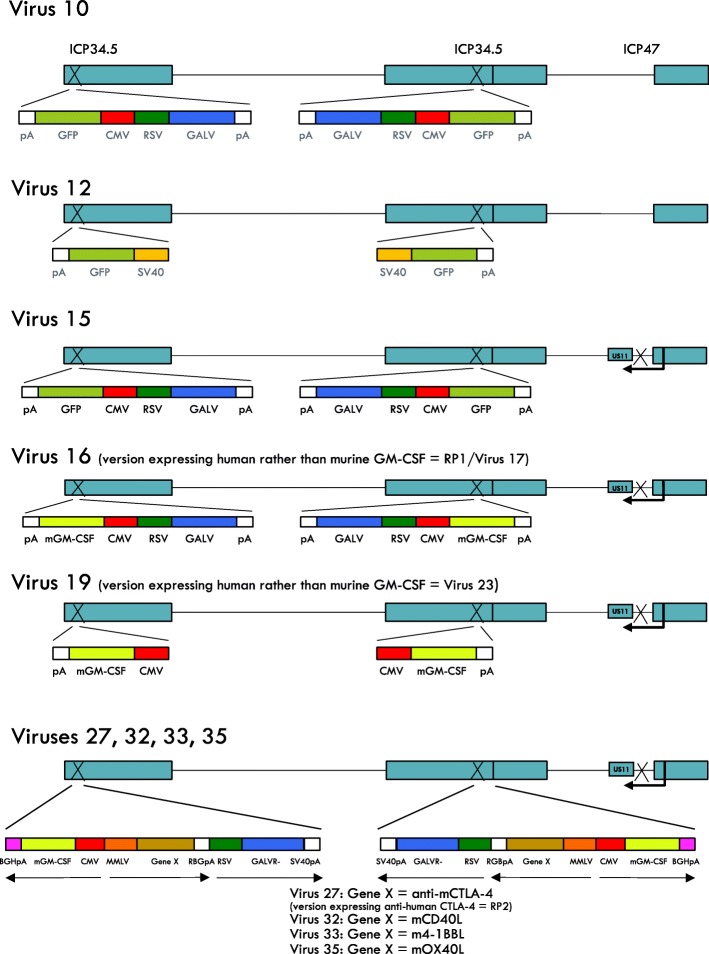
Schematic representation of the viruses constructed in this study. The genome structures of the viruses constructed and tested. The construction of each virus is described in detail in the Additional file 1

To render strain RH018 non-pathogenic and replication-selective for tumors, the HSV-1 genes encoding infected cell protein (ICP) 34.5 and ICP47 were deleted. ICP34.5, the so-called neurovirulence factor, has functions which include overcoming host anti-viral (i.e. interferon-mediated) responses which would otherwise block virus replication in normal tissue, and the expression of which is essential for pathogenicity [19, 20]. Deletion of ICP34.5 inhibits replication in normal tissue but ICP34.5 is dispensable for replication in tumors [14] by virtue of their generally having impaired interferon-mediated responses through various mechanisms [21]. ICP47 is an inhibitor of antigen presentation in HSV-1 infected cells [22], the deletion of which also increases the expression of the HSV US11 gene by placing the coding sequence for US11 adjacent to the immediate/early promoter for ICP47 [13]. US11 has functional redundancy with ICP34.5 and immediate/early expression of US11 restores to HSV-1 some level of resistance to interferon [23]. This increases replication in tumors, without reducing the tumor selectivity achieved through the deletion of ICP34.5 [13]. ICP34.5- and ICP34.5/47-deleted versions of HSV-1 have been extensively tested in clinical trials [24] and T-VEC (which has both the ICP34.5 and ICP47 deletions) is U.S. FDA approved for the treatment of advanced melanoma. In all cases, these viruses have been shown to be well tolerated, including through direct intracerebral injections in patients with glioma [25]. This proven safety and efficacy profile provided the basis for using the same disabling approach here. All viruses were generated by recombination of viral and plasmid DNA using standard methods, followed by clone selection based on the presence or absence of GFP [26]. The genome structures of the viruses constructed and tested in this paper are shown in (Fig. 1). The details of the construction of each virus is described in the Additional file 1.

### Augmenting the natural ability of HSV-1 to kill tumor cells

**Fig. 2:**
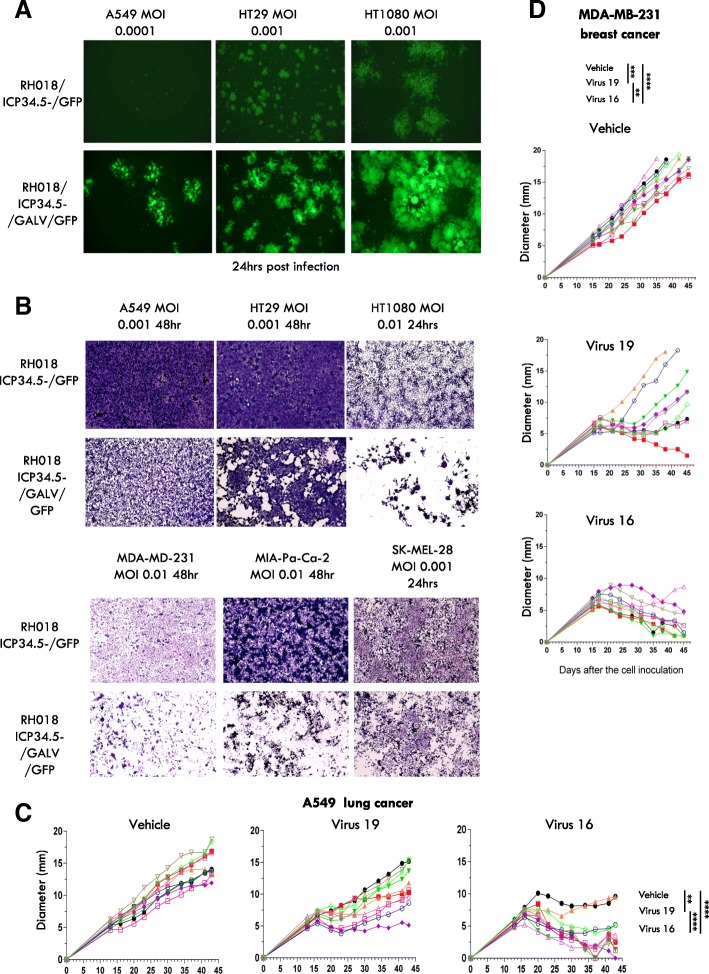
The effects of GALV-GP-R^−^ expression on human tumor cell lines in vitro and human tumor xenograft models in vivo. **a** Images of cell lines infected with Virus 12 (expresses GFP) top panel and (**a**) Images of cell lines infected with Virus 10 (expresses GFP and GALV-GP-R^−^). **b** Images representing the cell killing effects of Virus 12 and (**b**) Virus 10 in a panel of tumor cells. **c** Individual tumor growth curves from mice treated with either vehicle, Virus 19 (expresses mGM-CSF) or Virus 16 (expresses mGM-CSF and GALV-GP-R^−^) in the A549 lung cancer model and (**d**) in the MDA-MB-231 breast cancer model. The dose level of virus was in each case 5 × 10^3^ pfu in 50 μl given 3x every other day. Statistical differences between groups were measured by one-way ANOVA at day 41 for the A549 model and at day 38 for the MDA-MB-231 model. *p < 0.05, **p < 0.01, ***p < 0.001, ****p < 0.0001

In order to augment the natural ability of HSV-1 to kill tumor cells, a codon-optimized version of a potent fusogenic membrane glycoprotein (GP) from gibbon ape leukemia virus (GALV) was additionally encoded in the virus backbone. Here, the R sequence was deleted (R^−^), which provides constitutive fusion properties to the GALV-GP [14]. The initial viruses constructed to test this approach expressed either GFP or GFP together with GALV-GP R^−^ (Virus 10 and Virus 12) (Fig. 1), which were first tested on a range of tumor cell lines in vitro. This demonstrated that potent cell-to-cell fusion was achieved through the expression of GALV-GP-R^−^, and that the plaques generated by these viruses were greatly enlarged, as visualized by expression of GFP (Fig. 2a). Cell killing potency was also greatly increased, with substantially greater killing being achieved at equivalent virus doses through the expression of GALV-GP-R^−^ across multiple cell lines (Fig. 2b). Next, the effects of GALV-GP-R^−^ were assessed in human tumor models in nude mice in which A549 and MDA-MB-231 tumor cells were grown in the flanks of mice and various doses of the viruses were tested for their ability to treat these pre-existing tumors. Again, expression of GALV-GP-R^−^ was seen to significantly enhance anti-tumor activity (Fig. 2c-d), even when the viruses were used at low dose levels (data with the viruses used at a 5 × 10^3^ pfu dose level are shown).

As GM-CSF has been included in a number of oncolytic viruses in clinical trials where clinical activity has been demonstrated, a codon-optimized version of the gene for mGM-CSF was also encoded in the base platform virus constructed (Virus 16)(Fig. 1) into which further genes would then subsequently be inserted. GM-CSF was driven by a CMV promoter, and as for the GALV-GP-R^−^ encoding gene also inserted into the ICP34.5 locus. The function of GM-CSF is to aid in the maturation and function of dendritic cells (DC) and to enhance the activity of macrophages, intended to lead to enhanced anti-tumor immunity [27].

### Expression of GALV-GP-R^−^ by the virus further augmented immunogenic cell death in human and rat tumor cell lines

**Fig. 3:**
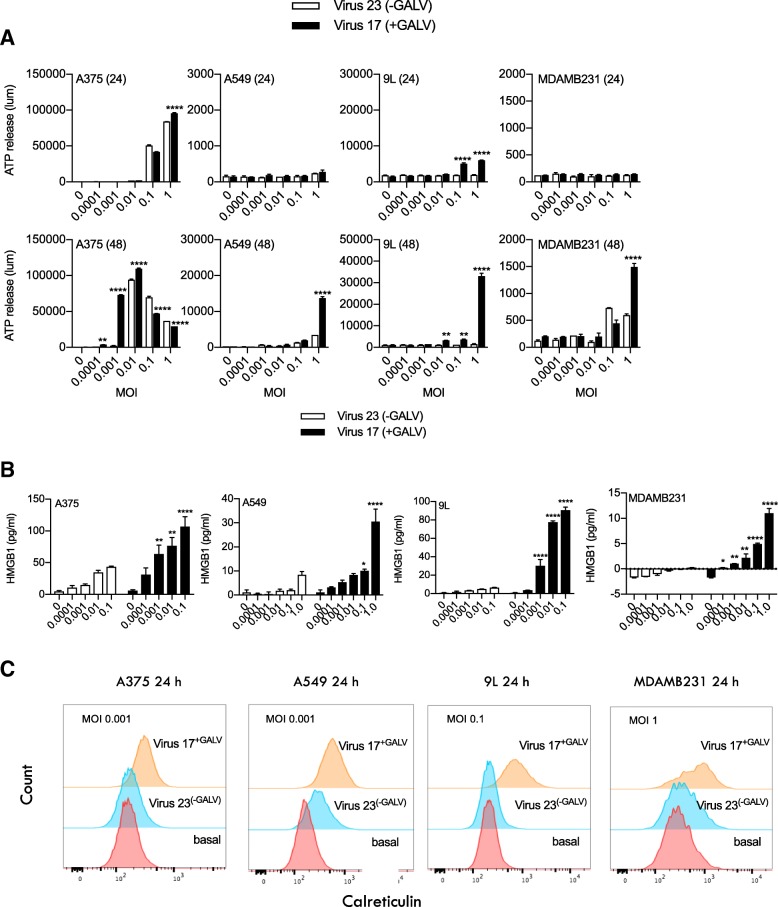
Markers of immunogenic cell death in cells treated with either Virus 23 (expresses hGM-CSF) or Virus 17 (expresses hGM-CSF and + GALV-GP R-) in vitro. **a** Levels of ATP release measured by luminescence in a panel of cell lines treated at the indicated MOI at 24 h post infection and (**a**) at 48 h post infection observed in cell-free supernatants treated with Virus 23 (indicated by the clear bars) and Virus 17 (indicated by the solid bars). **b** ELISA measuring HMGB1 (pg/ml) levels in cell-free supernatants from cells treated for 48 h with MOI 0.0001–1. **c** Histogram showing the expression levels of surface calreticulin (CRT) in cells treated at indicated MOI 0.01 for 48 h. Data show un-permeabilized, viable cells stained with CRT and measured by FACS. Statistical differences between groups were determined by using two-way ANOVA, *p < 0.05, **p < 0.01, ***p < 0.001, ****p < 0.0001

To test whether the expression of GALV-GP-R^−^ increased immunogenic cell death (ICD), A375, A549, 9 L and MDA-MB-231 tumor cell lines were treated with viruses with and without the insertion of the gene encoding GALV-GP-R^−^. After 24 or 48 h., cell supernatants and/or cell surfaces were assessed for levels of well characterized markers of ICD (ATP, HMGB1 and calreticulin) [28]. Infected cells demonstrated a dose-related increase in ATP from A375, A549, 9 L and MDA-MB-231 cells (Fig. 3a), and in HMGB1 from A375, A549, 9 L, and MDA-MB-231 cells (Fig. 3b), and also exhibited an increase in surface staining for calreticulin on A375, A549, 9 L, and MDA-MB-231 cells (Fig. 3c). All the ICD markers were substantially increased through the use of the virus expressing GALV-GP-R^−^ as compared to the otherwise equivalent virus which did not express GALV-GP-R^−^.

### Expression of GALV-GP-R^−^ further augments systemic anti-tumor effects

**Fig. 4:**
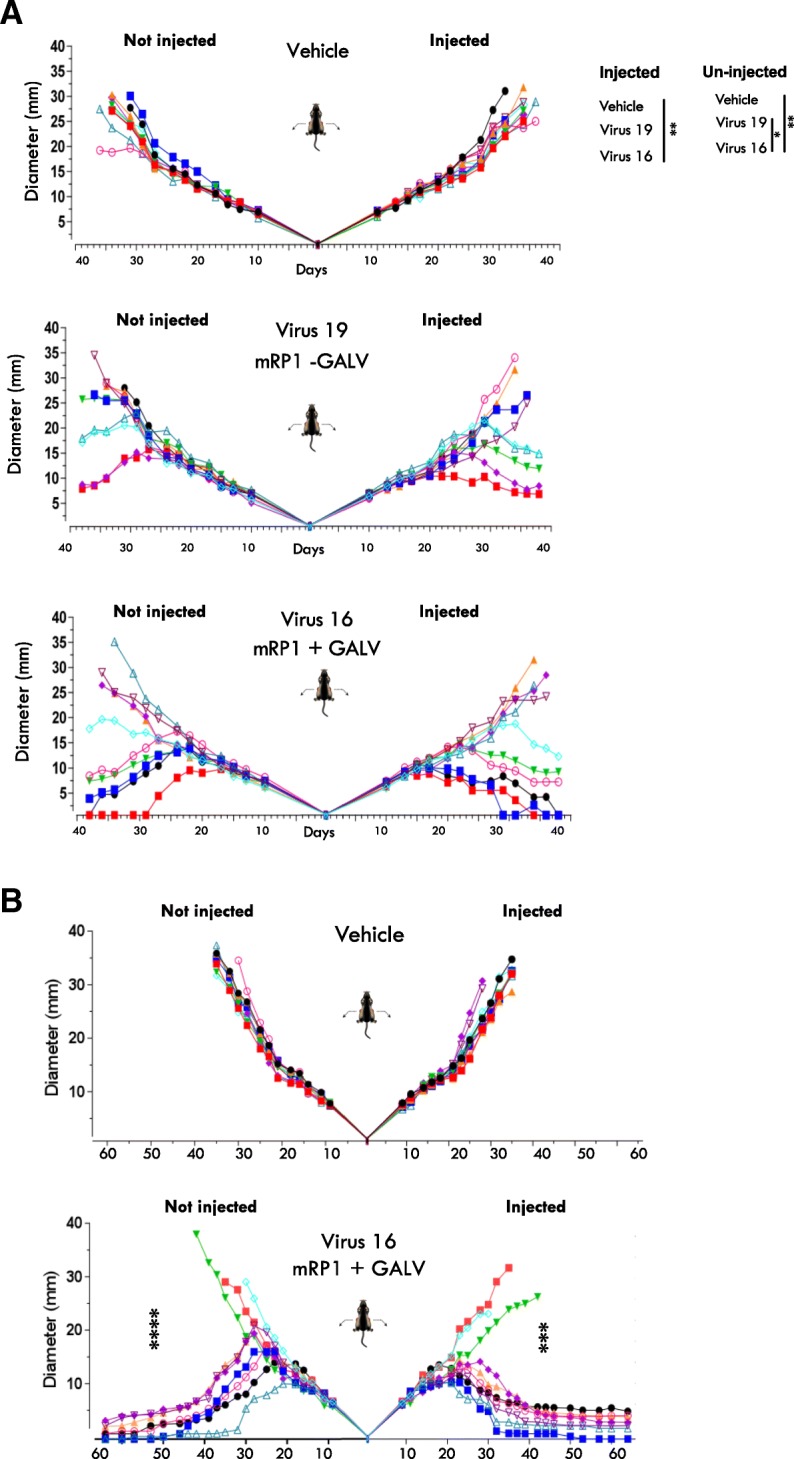
Effects of GALV-GP-R^−^ expression in an immune competent tumor model. **a** Tumor growth curves of rat 9 L tumors treated with either vehicle (PBS), Virus 19 (expresses mGM-CSF) or Virus 16 (expresses mGM-CSF and GALV-GP R^−^). Virus or vehicle were injected into the right tumor only. **b** A repeat of the experiment in (A), treating with either vehicle or Virus 16 but with longer follow up until day 60. 5 × 10^6^ pfu of virus in 50 μl was given 5x every other day in each case. Statistical differences between groups were measured by one-way ANOVA at day 31 for **a** and at day 35 for **b**. *p < 0.05, **p < 0.01, ***p < 0.001, ****p < 0.0001

While the receptor for GALV-GP, PiT1, is expressed in all mammalian cells [29], the murine version of PiT1 is incompatible with GALV-GP and no fusion (or infection with GALV itself) occurs. Rat PiT1 is, however, compatible with GALV-GP, and rats were therefore used to assess the effects of GALV-GP-R^−^ expression in an immune competent bilateral tumor model. Rats also allow larger tumors to be studied than is possible in mice, and murine GM-CSF is also partially functional in rats [30, 31]. Here, rat 9 L tumor cells were implanted in both flanks of rats and then tumors in the right flank were treated with a virus expressing only mGM-CSF (Virus 19) or expressing both mGM-CSF and GALV-GP R^−^ (Virus 16) (Fig. 4). In these experiments, tumor regression was seen in both injected and uninjected tumors with a significantly enhanced effect through the expression of GALV-GP-R^−^. While the experiment shown in Fig. 4a was sufficient to demonstrate these enhanced effects mediated through the expression of GALV-GP-R^−^, the study period was relatively short. As a result, a further experiment was conducted (Fig. 4b) where animals were treated with vehicle or Virus 16 and followed to 60 days. This demonstrated that both the injected and contralateral tumor in seven of ten animals completely regressed and that treated rats remained tumor free until the termination of the experiment.

### Treatment with virus 16 increases CD8+ T cell infiltration and PD-L1 expression levels in tumors

**Fig. 5:**
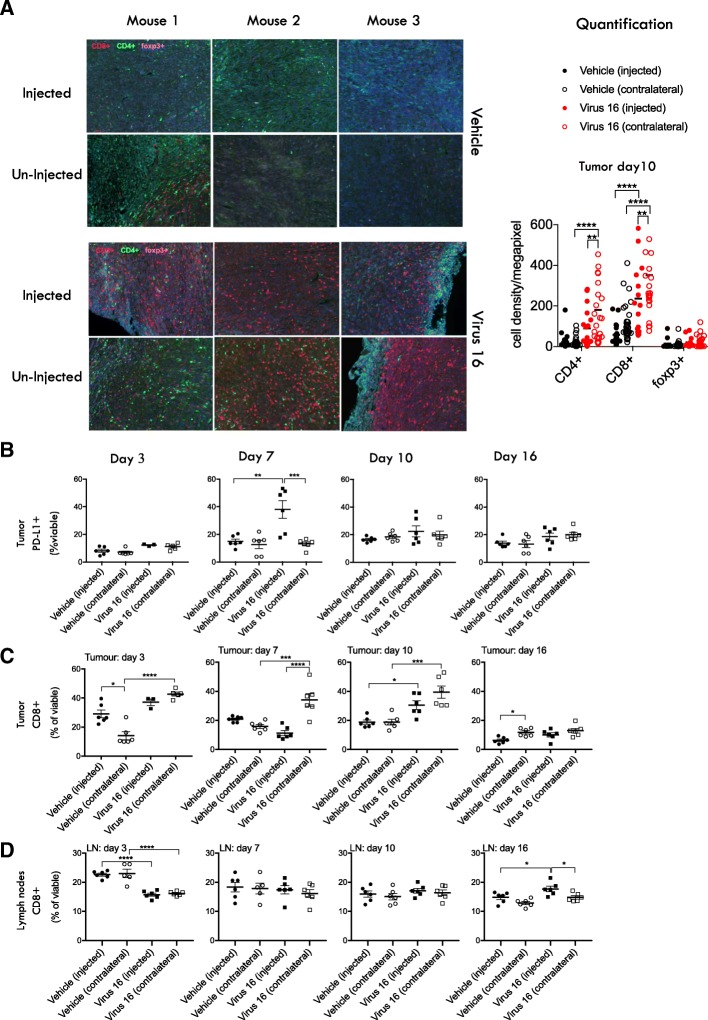
Tumors from Virus 16 treated animals demonstrate increased levels of CD8+ T cells and PD-L1*.* **a** Immunohistochemistry staining for CD8 (red), CD4 (green) and foxp3 (pink) of injected and un-injected 4434 tumors from mice either treated with mock or with Virus 16 (expresses mGM-CSF and GALV-GP R^−^) 10 days after treatment. **b** Relative frequency of PD-L1+ cells in mice bearing 4434 bi-flank tumours treated in the right flank with Virus 16 or vehicle on days 1, 3 and 5 and collected on days 3, 7, 10 and 16 after the first day of treatment. **c** The relative frequency of tumor infiltrating CD8+ cells, gated from the viable cell population, from tumours collected on days 3, 7, 10 and 16. **d** The relative frequency of CD8+ cells from lymph nodes at days 3, 7, 10 and 16. Statistical differences between groups were determined by using two-way ANOVA, *p < 0.05, **p < 0.01, ***p < 0.001, ****p < 0.0001

To confirm the hypothesis that local treatment with Virus 16 increases levels of infiltrating CD8+ T cells, bilateral 4434 tumors were established in the flanks of immune competent C57BL/6 J mice and treated as described in Methods with Virus 16 injected into the right flank tumor. Injected and un-injected tumors were harvested at 10 days following injection and assessed for the presence of CD8+ T cells, CD4+ T cells and CD4 + FoxP3+ regulatory T cells (Tregs) by immunohistochemical (IHC) assessment using the Vectra platform as previously described [16] (Fig. 5a). While there was only a limited effect on the level of CD4+ T cells and no obvious effects on Tregs, there was a significant increase in CD8+ T cells in both injected and contralateral uninjected tumours in virus-treated animals, but not in vehicle-treated controls (Fig. 5a). PD-L1 levels could not be assessed by IHC due to the inability to identify a suitable anti-mouse PD-L1 antibody for use in IHC. However, flow cytometry analysis demonstrated a significant increase in relative frequency of PD-L1 positive cells at day 7, predominantly in injected tumors in virus-treated animals but not in vehicle-treated controls (Fig. 5b). FACS analysis also demonstrated that the relative frequency of CD8+ T cells were significantly increased in the injected (ipsilateral) and un-injected (contralateral) tumors at day 10 (Fig. 5c). A significant increase in CD8+ T cells was also observed in the draining lymph nodes from injected tumors in treated mice at day 16 as compared to vehicle-treated control animals (Fig. 5d).

### Virus 16 productively replicates in injected tumors

To confirm that Virus 16 replicated in injected tumors, a time course experiment was conducted where mouse 4434 tumors were implanted in both flanks of mice as previously described. Virus 16 was injected into the right tumor and then left and right tumors harvested at 3, 7 and 10 days and the levels of live virus present per tumor determined by titration on BHK cells. This demonstrated that virus was detected at levels at least equal to the input level of virus until Day 7 in injected tumors and at lower levels in draining lymph nodes, but no virus was detected at any time in uninjected tumors (Additional file 1: Figure S2). This further confirmed that the virus remains localized to sites of injection and anenestic effects are due to immune-mediated systemic effects, and not due to the trafficking of virus from injected to uninjected contralateral tumors.

### Confirmation of synergy with immune checkpoint blockade

**Fig. 6:**
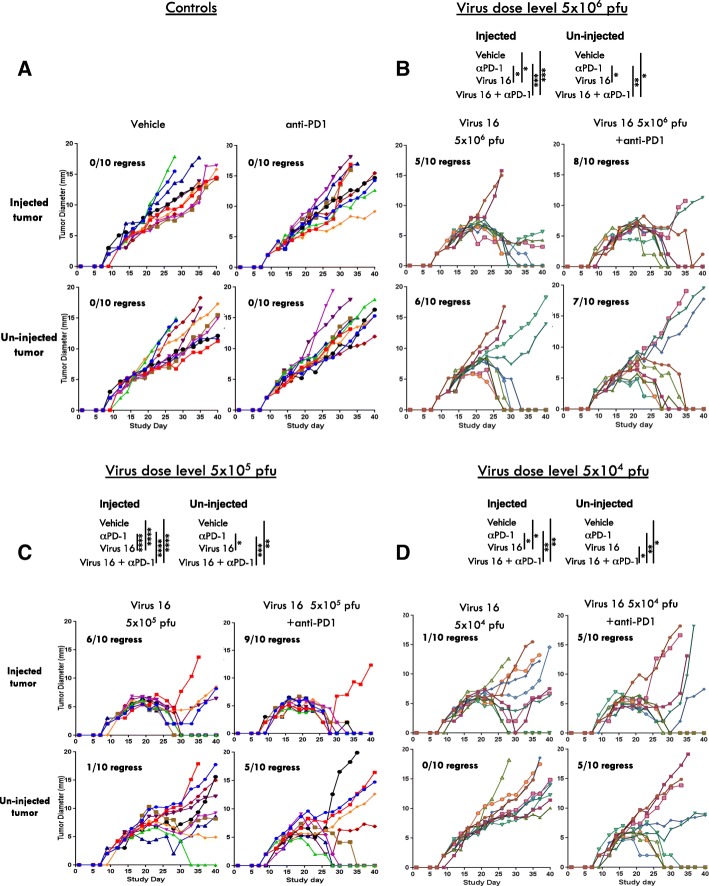
Effects of combination treatment with Virus 16 and anti-PD1. **a-d** Individual tumor growth curves of injected (right) and contralateral/un-injected (left) tumors from BALB/C mice bearing A20 lymphoma tumors treated with either (**a**) vehicle or anti-PD1, **b** Virus 16 (5 × 10^6^ pfu/dose 3x), or Virus 16 (5 × 10^6^ pfu/dose 3x) and anti-PD1, **c** Virus 16 (5 × 10^5^ pfu/dose 3x) or Virus 16 (5 × 10^5^ pfu/dose 3x) and anti-PD1 and (**d**) Virus 16 (5 × 10^4^ pfu/dose 3x) or Virus 16 (5 × 10^4^ pfu/dose 3x) and anti-PD1. Statistical differences between groups were measured by one-way ANOVA with multiple comparisions at day 28. *p < 0.05, **p < 0.01, ***p < 0.001, ****p < 0.0001

Based on prior evidence of clinical synergy between oncolytic therapy and immune checkpoint blockade [7, 8] and on the increased expression of PD-L1 induced by Virus 16, we sought to evaluate the effects of the combination of Virus 16 and PD-1 blockade in vivo*.* For this experiment the bilateral murine A20 lymphoma tumor model was used as these cells are susceptible to HSV-1, immune competent mice can be used, and anti-murine PD-1 antibodies are available to test the combination, although no GALV-GP R^−^ mediated effects will be seen. While anti-PD-1 treatment alone demonstrated minimal if any activity in this relatively anti-PD1 resistant model (Fig. 6a), enhanced anti-tumor effects were seen in both injected and uninjected tumors with Virus 16 in combination with anti-PD1 antibody therapy as compared to either therapy alone (Fig. 6b-d). These effects were most evident at low virus dose where the virus alone is least effective, and also particularly evident in uninjected tumors where the combination led to a significant enhancement of the anti-tumor effect (Fig. 6d). Figure 6b-d shows data using three dose levels of Virus 16 alone and in combination with murine anti-PD1, with in each case increased activity of the combination as compared to single agent treatment.

### Systemic effects can be further augmented through arming with anti-CTLA-4 or immune co-stimulatory pathway-activating ligands

**Fig. 7:**
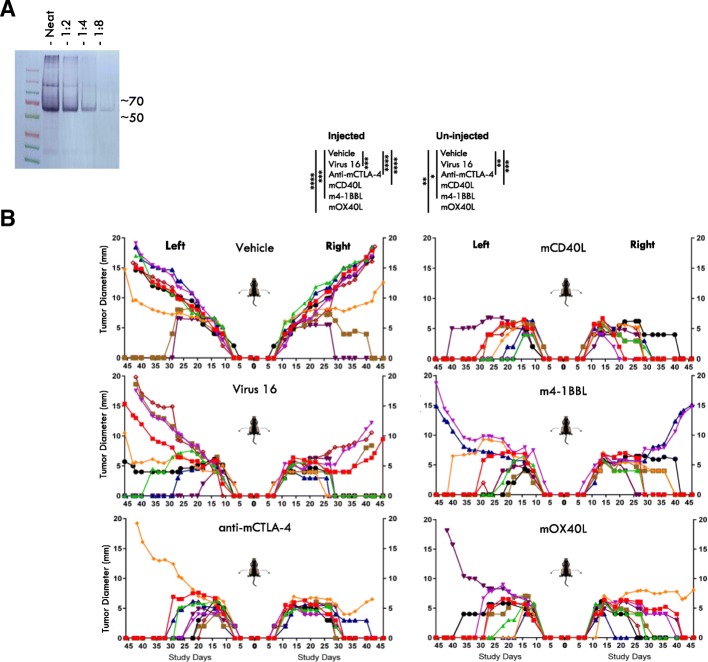
Expression of anti-CTLA-4 or immune co-stimulatory pathway activating ligands further increases the efficacy of Virus 16 in vivo. **a** Western blot showing the expression of of anti-mouse CTLA-4 as detected in cell lysates from cells infected with Virus 27. **b** Individual tumor growth curves of injected and contralateral tumors from BALB/C mice bearing A20 lymphoma tumors treated with either vehicle, Virus 16 (expresses GM-CSF and GALV-GP R-), Virus 27 (additionally expresses anti-mCTLA-4, Virus 32 (additionally expresses mCD40L), Virus 33 (additionally expresses m4-1BBL) or Virus 35 (additionaly expresses mOX40L). The dose level of virus was in each case 5 × 10^4^ pfu in 50 μl given 3x every other day. Statistical differences between groups were measured by one-way ANOVA at Day 40. *p < 0.05, **p < 0.01, ***p < 0.001, ****p < 0.0001

Virus 16, which expresses mGM-CSF and GALV-GP-R^−^, was then further developed to take advantage of the capacity of HSV-1 to encode other proteins intended to further augment the anti-tumor immune response. Thus, with these considerations in mind, to test these concepts Virus 16 was further engineered to express either an anti-mouse CTLA-4 antibody-like molecule or mouse CD40L, mouse OX40L or mouse 4-1BBL (Fig. 1). Following confirmation of expression by western blot analysis for anti-mouse CTLA-4 (Fig. 7a) and by ELISA for mCD40L, m4-1BBL and mOX40L (data not shown), these viruses were tested in the mouse bilateral A20 model, using a low virus dose (5 × 10^4^ pfu) which at that dose does not mediate a substantial anenestic effect in non-injected tumors for Virus 16. This demonstrated that virus-mediated delivery of each of these proteins was effective at increasing the antitumor effect, not only in injected tumors but more markedly in un-injected tumors (Fig. 7b). A further experiment where 15 mice previously cured of bilateral tumors were challenged with new tumor cells on the contralateral flank on day 108 demonstrated that these anti-tumor effects in combination with anti-PD1 are highly durable (Additional file 1: Figure S3A) and that due to the demonstrated protection of fourteen of the fifteen mice from re-challenge that effective memory immune responses had been induced. Anti-PD1 alone in this experiment had no significant anti-tumor effect (Additional file 1: Figure S3B).

## Discussion

In this study, we reported the development of a new oncolytic immunotherapy platform based on HSV-1 starting with a new clinical strain of HSV-1 isolated from an individual with herpes cold sores. This strain, RH018A was found to have broad lytic activity across a range of tumor cell lines in vitro. The new virus strain was developed for oncolytic use by deletion of the ICP34.5 and ICP47 encoding genes and insertion of a gene encoding the fusogenic protein GALV-GP-R^−^. HSV-1 is a naturally highly lytic virus which kills infected cells rapidly, and at low dose. Release of tumor antigens through this process would be expected to be highly immunogenic, but, even so, improvements to these properties would be beneficial. A number of fusogenic proteins, including from measles virus and a number of retroviruses have previously been tested in various gene therapy and oncolytic approaches to treating cancer, including when expressed from oncolytic HSV-1 [14]. However, while in all cases a high degree of anti-tumor efficacy was seen in pre-clinical models, with GALV-GP-R^−^ showing particular promise, no fusogenic approach to cancer therapy (oncolytic or otherwise) has previously progressed to clinical trials. GALV-GP-R^−^ kills cells by cell-to-cell membrane fusion (syncytium formation) following binding to the constitutively expressed PiT1 receptor for GALV [29]. This mechanism provides a large bystander effect around each infected cell, increasing the area of killing achieved. It has also been demonstrated that GALV-GP-R^−^-mediated cell death is highly immunogenic [14]. For these reasons, i.e. the potential to increase both the extent of tumor killing achieved and the immunogenicity of cell death, GALV-GP-R^−^ was included in the viruses developed here. GM-CSF was also included in the base platform virus from this program (Virus 16) since a number of oncolytic viruses in clinical trials have also encoded GM-CSF and clinical activity has been demonstrated, including talimogene laherparepvec which is U.S. FDA approved for the treatment of advanced melanoma [5], CG0070, an oncolytic adenovirus [32], and JX-594 (Pexavec; an oncolytic vaccinia virus [33].

GALV-GP-R^−^ expression was demonstrated to increase ICD in vitro as demonstrated by increased exposure of ecto-calreticulin and release of intracellular ATP and HMGB1. We also observed an increase in both local (i.e. injected or enestic) and systemic (i.e. uninjected or anenestic) [34] anti-tumor effects in unilateral mouse xenograft and/or bilateral tumors in immune competent rats. Increased CD8+ T cells and PDL1 expression levels in tumors were also observed. Consistent with the increased expression of PD-L1, we also found further improvement in therapeutic responses in combination with PD1 blockade in immune competent mice bearing established A20 tumors. In order to test the impact of GALV-GP-R^−^ in an immune competent host, we utilized the rat 9 L glioma model since rats, unlike mice, express a version of the PiT1 receptor which is compatible with GALV-GP-R^−^. In this model, Virus 16 also demonstrated significant tumor regression in both injected and uninjected tumors, which is particularly notable since the tumors in this model are substantially larger than is possible in mice. Collectively, these data support the clinical development of the human version of Virus 16, i.e. expressing human rather than mouse GM-CSF (Virus 17; RP1), which has entered clinical trials in a number of solid tumor types in combination with anti-PD1 therapy [35].

This prototype and initial clinical development candidate virus was then used as the basis from which to express further therapeutic genes aimed at amplifying the immune effects achieved. Here, the intention was to focus on the delivery of genes encoding proteins which exert their action at the site and time of immune response initiation (i.e. in injected tumors and draining lymph nodes), rather than through expression of proteins which would be required systemically to have their maximum effect. An example of the former includes CTLA-4 blockade, as CTLA-4 inhibits the induction of immune responses by competing with CD28 for binding to B7 on antigen presenting cells, making local intratumoral delivery an attractive option. An example of the latter would include anti-PD1 or -PD-L1 antibodies, since the PD1/L1 interaction inhibits the effector immune response systemically at the T cell/tumor interface, rendering local, virus-driven expression less appealing. Immune co-stimulatory pathway-activating proteins are also inviting candidates for intratumoral delivery, since they are expected to act at the site of immune response initiation to stimulate T cells. In addition, for these potentially toxic molecules, intratumoral delivery would restrict expression to the tumour compartment and might be expected to reduce the side effects that have occurred with systemic agonistic antibody-based approaches.

Initial viruses expressed an anti-CTLA-4 antibody-like molecule or immune co-stimulatory pathway-activating ligands (CD40L, 4-1BBL or OX40L). In each case, local delivery was demonstrated to increase the anenestic effect, validating the approach and providing further candidate viruses for clinical development. Overall, it is intended that the modular system developed will be used to express additional candidate therapeutic genes individually and in combination as promising candidate target pathways continue to be identified as the immune-oncology field matures.

## Conclusion

We report the development of a new oncolytic immunotherapy platform based on HSV-1 from the starting point a new clinical strain of HSV-1 isolated from an individual with herpes cold sores. This strain, RH018A, was further developed for oncolytic use by deletion of the ICP34.5 and ICP47 encoding genes and insertion of a gene encoding the fusogenic protein GALV-GP-R^−^. This led to an increase in the direct oncolytic effect and in immunogenic cell death in vitro. Treatment of xenograft models demonstrated that GALV-GP R^−^ enhanced direct tumor killing in-vivo and treatment of a syngeneic tumor model demonstrated enhanced anenestic responses. As expected, treatment effects were further improved in combination with PD1 blockade. This platform virus, which also expressed GM-CSF, was then used to express further therapeutic genes aimed at amplifying the immune effects achieved, an anti-CTLA-4 antibody-like molecule, or immune co-stimulatory pathway-activating ligands (CD40L, 4-1BBL or OX40L). In each case, local delivery was demonstrated to increase the anenestic effect, validating the approach and providing further candidate viruses for clinical development.

## Additional file

Additional file 1: Swab samples, Screening of the virus strains, Plasmid construction, Virus construction. **Figure S1.** Comparison of the tumor cell killing ability of clinical strains of HSV. **Figure S2.** Virus 16 can be retrieved in injected tumors but not contralateral 4434 tumors by TCID50 assay. **Figure S3.** Mice treated with Virus 16 and anti-PD1 exhibit long-term survival and reject subsequent tumor re-challenge. (DOCX 15807 kb)

## Data Availability

The data set analyzed for the current study is available from the corresponding author on reasonable request.

## Abbreviations

CTLA4: Cytotoxic T-lymphocyte associated protein 4
GM-CSF: Granulocyte macrophage colony stimulating factor
HSV: Herpes simplex virus
ICP: Infected cellular protein
MOI: Multiplicity of infection
OV: Oncolytic virus
PD1: Programmed cell death protein 1
