# Supplementary material for: Development of a new fusion-enhanced oncolytic immunotherapy platform based on herpes simplex virus type 1
Source: J Immunother Cancer. 2019 Aug 10;7:214. doi: 10.1186/s40425-019-0682-1 (PMC6689178; doi:10.1186/s40425-019-0682-1)
Supplement: Supplementary file 1 — Swab samples, Screening of the virus strains, Plasmid construction, Virus construction. Figure S1. Comparison of the tumor cell killing ability of clinical strains of HSV. Figure S2. Virus 16 can be retrieved in injected tumors but not contralateral 4434 tumors by TCID50 assay. Figure S3. Mice treated with Virus 16 and anti-PD1 exhibit long-term survival and reject subsequent tumor re-challenge. (DOCX 15807 kb) [file 40425_2019_682_MOESM1_ESM.docx]

**Additional file 1**

**Supplementary methods**

Swab samples

Screening of the virus strains

Plasmid construction

Virus construction

**Supplementary Figures:**

Suppl Fig.1

Suppl Fig.2

Suppl Fig.3

**Supplementary methods**

**Swab samples**

Swabs taken from cold sores from otherwise healthy volunteers were used to infect BHK 21 C13 cells (ECACC 85011433). Samples that generated cytopathic effect (CPE) within 48 hours were confirmed to be herpes simplex virus type1 (HSV-1) by immunofluorescence using an anti HSV-1 antibody (Abcam Cat No: ab9533) and also by PCR using primers that amplify the HSV-1 RL1 (ICP34.5) region (nucleotides 513-1540 and 124834-125861 from NCBI Reference Sequence: NC_001806.2) and the US12 (ICP47) region (nucleotides 145316-145582).

**Screening of the virus strains**

Two rounds of virus screening were conducted. In the initial screen, stocks from 29 virus samples were used to infect a range of human cancer cell lines in subsets of 5-8 virus strains per experiment. The cell lines used were Fadu (ATCC® HTB-43™), SK-MEL-28 (ATCC® HTB-72™), A549 (ECACC 86012804), HT1080 (ECACC 85111505), miaPaCa-2 (ECACC 85062806), HT29 (ECACC 91072201) and MDA-MB-231 (ECACC 92020424). The cells were infected using a range of multiplicities of infection (MOI) from 0.001 to 0.1. The infected cell monolayers were fixed and then stained with crystal violet at 24 hr and 48 hr post infection. The plates were then visually inspected and the virus strains ranked within each subset by two independent observers for their cell killing ability. The scores from each cell line were used to generate an average score for each virus strain. A low total score therefore indicated a greater ability to kill across the panel of cell lines as compared to a higher score. Using these scores the top 8 virus strains where selected for a second round of screening using the same method to identify the strain to be used for further development.

**Plasmid construction**

All of plasmids used were generated by Genscript Inc by a combination of gene synthesis using sequence information deposited in Genbank and subcloning.

**Virus construction**

All viruses were generated by recombination of viral and plasmid DNA using standard methods, followed by clone selection based on the presence or absence of GFP (23). Viral DNA was obtained from infected BHK cells using DNAzol (Thermo Fisher). For deletion of ICP34.5, nucleotides 123464–124953 and 125727-126781 (from NCBI NC_001806.2) were inserted into plasmid pSP72 (Promega) to generate plasmid pΔICP34.5. GFP under the control of the CMV promoter and BGH pA and GALV-GP-R- under the control of the RSV promoter and SV40pA was inserted into pΔICP34.5 to generate plasmid pΔICP34.5/GALVR-/GFP. Virus 10 (18/34.5-/GFP/GALV/34.5) was generated by recombination of strain RH018 DNA and plasmid pΔICP34.5/GALVR-/GFP. GFP under the control of the CMV promoter and BGH pA was inserted into pΔICP34.5 to generate plasmid pΔICP34.5/CMV/GFP. Virus 12 (18/34.5-/GFP/34.5) was generated by recombination of strain RH018 DNA and plasmid pΔICP34.5/CMV/GFP.

For deletion of ICP47, nucleotides 143680–145300 and 145582–147083 (from NCBI NC_001806.2) were inserted into plasmid pSP72 (Promega) to generate plasmid pΔICP47. GFP under the control of the CMV promoter and BGH pA was inserted into pΔICP47 to generate plasmid pΔICP47/CMV/GFP. Virus 11 (18/47-/GFP/47) was generated by recombination of strain RH018 DNA and plasmid pΔICP47/CMV/GFP. GFP was removed from by recombination of Strain 11 DNA and plasmid pΔICP47 to generate Virus 13.

Virus 15 (18/47-/34.5-/GFP/GALV/34.5) was generated by recombination of Virus 13 DNA and plasmid pΔICP34.5/GALVR-/GFP. The GFP sequence in plasmid pΔICP34.5/GALVR-/GFP was replaced with the sequence for mouse GM-CSF to generate plasmid pΔICP34.5/GALVR-/mGM-CSF. Virus 16 (18/47-/34.5-/mGM-CSF/GALV/34.5) was generated by recombination of Virus 15 DNA with plasmid pΔICP34.5/GALVR-/mGM-CSF. The GFP sequence in plasmid pΔICP34.5/CMV/GFP was replaced with the sequence for mouse GM-CSF to generate plasmid pΔICP34.5/GALVR-/mGM-CSF. Virus 19 (18/47-/34.5-/mGM-CSF/34.5) was generated by recombination of Virus 15 DNA and plasmid pΔICP34.5/mGM-CSF.

GFP under the control of the MMLV LTR promoter and RBGpA was inserted into plasmid pΔICP34.5/GALVR-/mGM-CSF to give plasmid pGALVR-/mGM-CSF/MMLV-GFP. Recombination of DNA from Virus 16 and plasmid pGALVR-/mGM-CSF/MMLV-GFP was used to generate Virus 25 (18/47-/34.5-/mGM-CSF/MMLV-GFP/ GALV/34.5). Virus 25 was subsequently used to generate Virus 27.

Virus 27 (18/47-/34.5-/mGM-CSF/MMLV-amCTLA4/GALV/34.5) was generated by recombination of Virus 25 and plasmid 119 (pGALVR-/mGM-CSF/MMLV-amCTLA-4). amCTLA-4 antibody expression is under the control of the MMLV LTR promoter and RBGpA. The anti-mouse CTLA-4 molecule comprised the variable regions from the 9D9 antibody (ref) linked together and fused to mouse IgG1.

Virus 32 (18/47-/34.5-/mGM-CSF/MMLV-mCD40L/GALV/34.5) was generated by recombination of Virus 25 and plasmid 79 (pGALVR-/mGM-CSF/MMLV-mCD40L). Mouse CD40L expression is under the control of the MMLV LTR promoter and RBGpA.

Virus 33 (18/47-/34.5-/mGM-CSF/MMLV-m41BBL/GALV/34.5) was generated by recombination of Virus 25 and plasmid 81 (pGALVR-/mGM-CSF/MMLV-m4-1BBL). Mouse 41BBL expression is under the control of the MMLV LTR promoter and RBGpA.

Virus 35 (18/47-/34.5-/mGM-CSF/MMLV-mOX40L/GALV/34.5) was generated by recombination of Virus 25 and plasmid 85 (pGALVR-/mGM-CSF/MMLV-mOX40L). Mouse OX40L expression is under the control of the MMLV LTR promoter and RBGpA.

**Supplementary Figures 1 - 3**

**Suppl. Fig 1. Comparison of the tumor cell killing ability of clinical strains of HSV.**

**(A)** Comparison of the nine HSV-1 isolates at the indicated multiplicity of infection (MOI) allowing the generation of a rank order of the top five isolates across the tumor cell line panel. Red boxes represent the virus stain ranked first on the indicated cell line, blue boxes represent the virus stain ranked second on the indicated cell line. **(B)** Comparison of strain RH018A with a representative ‘average’ strain from the screen (Strain RH065A). Representative data at only an individual MOI and time point in each case are shown.

**Suppl Fig. 2 Virus 16 can be retrieved in injected tumors but not contralateral 4434 tumors.**

TCID50 assays showing the viral titers of HSV-1 from tumors, lymph nodes and spleen from mice bearing 4434 tumors treated with vehicle or PBS at days 3, 7 and 10.

**Suppl Fig.3: Mice treated with virus 16 and anti-PD1 exhibit long-term survival and reject subsequent tumor re-challenge.**

**(A)** Individual tumor growth curves from 15 mice achieving complete tumor regression following treatment with Virus 16 and anti-PD1 which were then re-challenged with new tumor cells on the contralateral flank at day 108. The right panel shows tumor growth in control re-challenge mice. **(B)** Individual tumor growth curves from mice treated with the anti-PD1 antibody alone.
